# Variance of age-specific log incidence decomposition (VALID): a unifying model of measured and unmeasured genetic and non-genetic risks

**DOI:** 10.1093/ije/dyad086

**Published:** 2023-06-22

**Authors:** John L Hopper, James G Dowty, Tuong L Nguyen, Shuai Li, Gillian S Dite, Robert J MacInnis, Enes Makalic, Daniel F Schmidt, Minh Bui, Jennifer Stone, Joohon Sung, Mark A Jenkins, Graham G Giles, Melissa C Southey, John D Mathews

**Affiliations:** Centre for Epidemiology and Biostatistics, Melbourne School of Population and Global Health, University of Melbourne, Melbourne, VIC, Australia; Centre for Epidemiology and Biostatistics, Melbourne School of Population and Global Health, University of Melbourne, Melbourne, VIC, Australia; Centre for Epidemiology and Biostatistics, Melbourne School of Population and Global Health, University of Melbourne, Melbourne, VIC, Australia; Centre for Epidemiology and Biostatistics, Melbourne School of Population and Global Health, University of Melbourne, Melbourne, VIC, Australia; Centre for Epidemiology and Biostatistics, Melbourne School of Population and Global Health, University of Melbourne, Melbourne, VIC, Australia; Genetic Technologies Ltd., Fitzroy, VIC, Australia; Centre for Epidemiology and Biostatistics, Melbourne School of Population and Global Health, University of Melbourne, Melbourne, VIC, Australia; Cancer Epidemiology Division, Cancer Council Victoria, Melbourne, VIC, Australia; Centre for Epidemiology and Biostatistics, Melbourne School of Population and Global Health, University of Melbourne, Melbourne, VIC, Australia; Centre for Epidemiology and Biostatistics, Melbourne School of Population and Global Health, University of Melbourne, Melbourne, VIC, Australia; Faculty of Information Technology, Monash University, Clayton, VIC, Australia; Centre for Epidemiology and Biostatistics, Melbourne School of Population and Global Health, University of Melbourne, Melbourne, VIC, Australia; School of Population and Global Health, University of Western Australia, Perth, WA, Australia; Division of Genome and Health Big Data, Department of Public Health Sciences, Graduate School of Public Health, Seoul National University, Seoul, Korea; Centre for Epidemiology and Biostatistics, Melbourne School of Population and Global Health, University of Melbourne, Melbourne, VIC, Australia; Cancer Epidemiology Division, Cancer Council Victoria, Melbourne, VIC, Australia; Cancer Epidemiology Division, Cancer Council Victoria, Melbourne, VIC, Australia; Precision Medicine, School of Clinical Sciences at Monash Health, Monash University, Clayton, VIC, Australia; Centre for Epidemiology and Biostatistics, Melbourne School of Population and Global Health, University of Melbourne, Melbourne, VIC, Australia

**Keywords:** Breast cancer, familial cause, familial odds ratio, familial risk ratio, genetic cause, genomic cause, major gene, non-familial cause, polygenic risk score, variance components

## Abstract

**Background:**

The extent to which known and unknown factors explain how much people of the same age differ in disease risk is fundamental to epidemiology. Risk factors can be correlated in relatives, so familial aspects of risk (genetic and non-genetic) must be considered.

**Development:**

We present a unifying model (VALID) for variance in risk, with risk defined as log(incidence) or logit(cumulative incidence). Consider a normally distributed risk score with incidence increasing exponentially as the risk increases. VALID’s building block is variance in risk, Δ^2^, where Δ = log(OPERA) is the difference in mean between cases and controls and OPERA is the odds ratio per standard deviation. A risk score correlated *r* between a pair of relatives generates a familial odds ratio of exp(*r*Δ^2^). Familial risk ratios, therefore, can be converted into variance components of risk, extending Fisher’s classic decomposition of familial variation to binary traits. Under VALID, there is a natural upper limit to variance in risk caused by genetic factors, determined by the familial odds ratio for genetically identical twin pairs, but not to variation caused by non-genetic factors.

**Application:**

For female breast cancer, VALID quantified how much variance in risk is explained—at different ages—by known and unknown major genes and polygenes, non-genomic risk factors correlated in relatives, and known individual-specific factors.

**Conclusion:**

VALID has shown that, while substantial genetic risk factors have been discovered, much is unknown about genetic and familial aspects of breast cancer risk especially for young women, and little is known about individual-specific variance in risk.

Key Messages

- Risk can be defined as age-specific log(incidence) or cumulative risk.
- The key metric for defining the risk discrimination of a risk factor is Δ = the log of the change in odds ratio per standard deviation of a possibly adjusted and transformed risk score with unit variance.
- Δ = the difference between cases and controls in mean risk score.
- Δ^2^ = the variance in risk attributed to this risk score.
- We show how variation in risk can be partitioned into measured and unmeasured genetic and non-genetic components.
- Variation in genetic risk is finite and its upper limit can be determined from the disease association (specifically the familial odds ratio which approximates the familial risk ratio for most diseases) within genetically identical (monozygotic: MZ) twin pairs.
- Genetic factors will not be important for risk prediction if the MZ twin pair odds ratio is weak, irrespective of disease frequency.
- Variation in non-genetic risk is unlimited.

## Background

A fundamental issue for epidemiology is the extent to which known and unknown factors explain how much people of the same age differ from one another in their disease risk. Given that risk factors can be correlated in relatives, familial risk factors—both (germline) genetic and non-genetic (e.g. shared environment)—must be considered.

This paper introduces a unifying model called Variance of Age-specific Log Incidence Decomposition (VALID), with risk defined as the age-specific log(incidence) or logit(cumulative incidence). Variance in risk as a quantitative trait is the building block. VALID considers familial and non-familial, genetic and non-genetic, measured and unmeasured variance in risk. It therefore brings together individual-specific and familial risks, including lifestyle, polygenes, major genes and shared environment, known and unknown.

VALID is in part based on Fisher’s seminal 1918 paper^1^ that introduced the concept of unmeasured genetic and non-genetic causes of variation in measured quantitative outcomes (traits); see Historical context in the Supplementary Material (available as Supplementary data at *IJE* online). Fisher warned that the concept of ‘heritability’ could be misleading,^2^ as we found when studying large immigrant and non-immigrant sibships.^3^ Here we essentially extend Fisher’s model to disease risk, and thereby to binary traits in general. Whereas Fisher converted familial correlations into variances in measured quantitative traits, VALID converts familial odds ratios into variances in risk.

## Modelling genetic and non-genetic familial and non-familial causes of variation

For a trait with total variance σ^2^ and an additive genetic component with variance A, the trait correlation is r_MZ_ = A/σ^2^ for monozygotic (MZ) twin pairs, r_DZ_ = ½A/σ^2^ for dizygotic (DZ) twin pairs and other first-degree relatives, ¼A/σ^2^ for second-degree relatives, and so on.^1^ This model was extended to include environmental (i.e. non-genetic) causes shared by (or common to) relatives, whose variance has historically been denoted by C. The classic twin model assumes C is the same for MZ and DZ pairs, so A = 2(r_MZ_ − r_DZ_)σ^2^ and C = (2r_DZ_ − r_MZ_)σ^2^ provided 2r_DZ_> r_MZ_ (Falconer’s formula).^4^ Under a flexible parametrization fitted using, for example, the multivariate normal model,^5^^,^^6^ the model can be extended to families, the genetic component can be modelled as a function of measured factors either as fixed or as random effects,^7^ and the shared environmental variance component, C, can take into account factors such as the extent to which pairs of relatives cohabit, have cohabited or have lived apart; see Modelling of the familial causes of variance in risk below.

## Development

### Risk score versus risk factor

**Figure 1:**
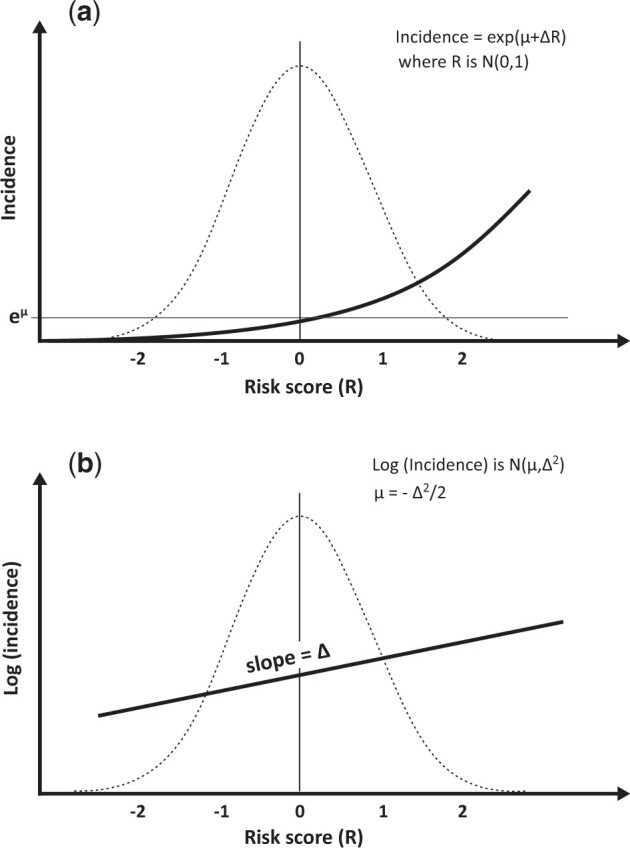
Incidence as an exponential function (a) and log(incidence) as a linear function (b) of the risk score under the VALID model for a risk score with a standard normal distribution, superimposed on the risk score’s density function (dotted line)

We represent a risk factor (which might be a composite of risk factors such as genetic markers) as a risk score that has a standard normal distribution, that disease incidence increases exponentially as the risk score increases (see Figure 1a), for which log(incidence) increases linearly as the risk score increases (see Figure 1b), at least when incidence is small (see below).

These characteristics have been observed for the combined associations of common genetic variants on risk of breast cancer based on additivity on the log risk scale both within and between markers to create an ‘additive’ polygenic risk score.^8^ This model is also inherent to case-control and cohort study analyses using logistic and Cox regression, respectively; see ‘Why log(incidence)?’ in the Conclusion.

We are studying variation in relative risk, not absolute risk per se, so the risk score must be adjusted for age and possibly other covariates, as should be standard practice in epidemiology. This approach underlies the odds ratio per adjusted standard deviation (OPERA) concept as a population measure of risk discrimination.^9^ For concreteness, we take risk to mean the log(incidence), although the VALID concept also applies to log(odds ratio) = logit(odds) and therefore to cumulative risk (e.g. lifetime risk or risk to a given age) or any binary trait in general. Our main interest is in diseases, not common traits.

VALID essentially follows models by us and others,^10–15^ except that here we assume the risk score has been standardized to have unit variance. This is important when interpreting the term ‘risk score’. Pharoah and colleagues^12^ and Clayton^13^ refer to the polygenic risk score, R, as having a log-normal distribution such that log(R) = Y is distributed as N(µ, σ^2^). VALID considers Z = (Y−µ)/σ, which has a standard normal N(0,1) distribution. The difference between cases and controls in mean Y is σ^2^;^12^ so the difference between cases and controls in mean Z is σ.

### Parameterization

**Figure 2:**
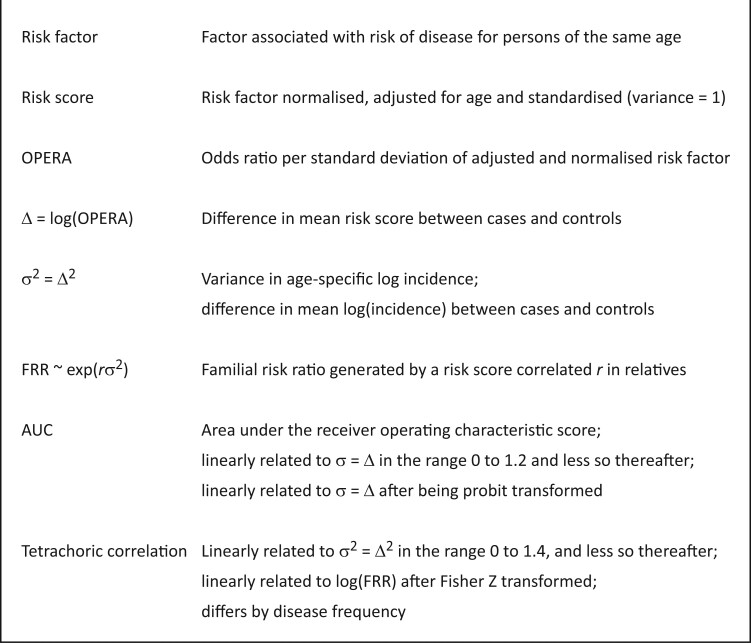
Definitions, descriptions, and relationships between major concepts underlying the VALID model

Figure 2 shows the key parameters involved in the VALID model. The strength of a risk score, in terms of its ability to differentiate cases from appropriate controls on a population basis, is assessed by log(OPERA), where OPERA is the odds ratio per adjusted standard deviation. The adjusted standard deviation is the standard deviation of the residuals after the risk factor has been adjusted for age and potentially other measures.^9^^,^^16^^,^^17^ Given that what is estimated for an adjusted risk factor is the change in risk per unit change of the risk factor, while conceptually holding constant all those measures taken into account by sampling and analysis, it is not appropriate to use the odds ratio per ‘unadjusted’ standard deviation.

Consider a risk score that is normally distributed for both cases and controls, and with the same variance in these two groups which, without loss of generality, we take to be 1. Let Δ = the difference between cases and controls in mean risk score. Then:


(1) Δ= log (OPERA).

(see Relationship between OPERA and Δ in the Supplementary Material, and in the Supplementary Material (available as Supplementary data at *IJE* online) in Schmidt DF and colleagues^18^).

Figure 1b shows the linear relationship between log(incidence) and the standardied risk score where log(incidence) has a normal distribution with mean µ and variance Δ^2^.

**Figure 3:**
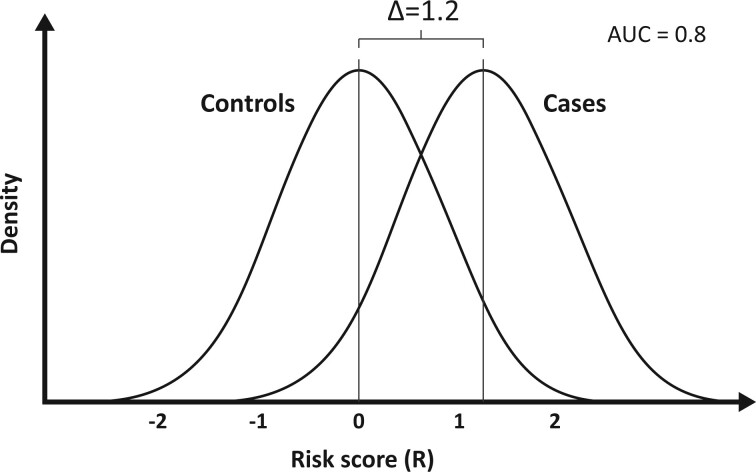
Density of the risk score distribution under the VALID model for cases and controls when Δ = 1.2 and the area under the receiver operating characteristic curve (AUC) = 0.8

There is a simple relationship between Δ = log(OPERA) and the area under the receiver operating characteristic curve (AUC) given by:


(2) $$\text{AUC}=\Phi \left(\Delta /\sqrt{2}\right),$$

where Φ is the cumulative distribution function of the standard normal distribution (see Relationship between AUC and Δ = log(OPERA) in Supplementary Material^18^). Therefore Δ = log(OPERA) is linearly related to probit transformed AUC irrespective of the disease prevalence. It is the difference between cases and controls in the mean of the standardized risk score and is also referred to in different ways in different disciplines, such as Cohen’s D.^19^Figure 3 shows the distribution of log(incidence) for cases and controls in the situation where Δ  =  1.2 and AUC = 0.8.

The variance of the log(incidence) is:


(3) $$\sigma^{2}=\Delta^{2}={\left[\text{log}\;\left(\text{OPERA}\right)\right]}^{2}$$

and is the square of the difference in means between cases and controls on the standardized risk score scale, the difference in mean log(incidence) between cases and controls, and the square of the logarithm of the odds ratio per standard deviation of the risk score; see Figure 2.^12^

### Familial risk caused by familial aspects of a risk factor: unifying equation

For a given pair of relatives, rel = twin pairs, siblings, etc, let the familial odds ratio be the odds of disease for the relative of an affected person divided by the odds of disease for the same type of relative of an unaffected person. A risk factor with a correlation in risk score between relatives of r_rel_, and a risk gradient of Δ = log(OPERA), generates a corresponding:


(4) $$\text{familial odds ratio}=\;\text{exp}(r_{\text{rel}}\Delta^{2}).$$

Given we are interested in diseases (see above), the familial odds ratio is approximately equal to the familial risk ratio (FRR_rel_) = the risk of disease for the unaffected relative of an affected person divided by the risk for the same type of unaffected relative of an unaffected person. In this setting:


(5) $${\text{FRR}}_{\text{rel}}∼\;\;\text{exp}(r_{\text{rel}}\Delta^{2}).$$

Once the relationship between Δ and the interquantile risk ratio is understood (see Relationship between IQRR and Δ in Supplementary Material,^18^) it can be seen that Equation (5) was in effect derived by Aalen^10^ under the assumption of a multiplicative risk and a ‘rare’ disease. For a polygenic model, Equation (4) was derived by Pharoah and colleagues^12^ and Clayton proved it was a good approximation for both the multiplicative and logistic risk models.^13^Equation (4) had previously been shown to apply to specific instances by Hopper and Carlin.^11^

We refer to Equation (4) as the Unifying Equation. It is fundamental to genetic epidemiology and plays a critical role in VALID because it allows the familial aspects of any risk factor to be interpreted in terms of its contribution to the disease association for all pairs of relatives. For diseases, Equation (5) implies that:


(6) $$\Delta ={\left[\text{log}\;\left({\text{FRR}}_{\text{rel}}\right)/r_{\text{rel}}\right]}^{0.5}$$

and from (2) and (5),


(7) $$\text{AUC}=\Phi {\left\{\left[\text{log}\;\left({\text{FRR}}_{\text{rel}}\right)/{2r}_{\text{rel}}\right)\right]}^{0.5}\}.$$

If the only cause of familial risk is genetic factors such that, for first degree-relatives, r_rel_ = 0.5, then:


(8) $$\text{AUC}=\Phi \{{\left[\text{log}\;\left({\text{FRR}}_{\text{rel}}\right)\right]}^{0.5}\}.$$

Under this assumption, if the FRR for first degree relatives is 2, then the maximum AUC that can be achieved by knowing all additive genetic factors is 0.80, corresponding to Δ  =  1.2 and σ^2^ = 1.4; see Figure 3.

Table 1 shows the different risk discrimination parameters for a selection of values across their ranges sufficient to allow for reasonably accurate interpolation.

**Table 1:** Comparative tabulation of different parameters of risk discrimination

AUC^a^	OPERA^b^	Δ^c^	Δ^**2**^^d^	FRR_MZ_^e^	IQRR^f^	UQRR^g^
0.50	1	0	0	1	1	1
0.55	1.2	0.18	0.03	1.04	1.6	1.2
0.60	1.4	0.36	0.13	1.14	2.5	1.5
0.65	1.7	0.54	0.30	1.34	4.0	1.8
0.70	2.1	0.74	0.55	1.74	6.7	2.1
0.75	2.6	0.95	0.91	2.50	12	2.4
0.80	3.3	1.19	1.42	4.12	22	2.8
0.85	4.3	1.47	2.15	8.58	49	3.1
0.90	6.1	1.81	3.28	26.8	135	3.5
0.95	10	2.33	5.41	224	706	3.8

a Area under the receiver operating characteristic curve (AUC).

b Odds ratio per standard deviation of the adjusted risk factor (OPERA).

c Difference in mean between cases and controls (Δ = log(OPERA)).

d Variance in log(incidence) (Δ^2^).

e Familial risk ratio if *r *=* *1.0 (FRR_MZ_ = exp(Δ^2^)).

f Interquartile risk ratio (IQRR).

g Upper-quartile risk ratio to the population average (UQRR).

### Modelling the familial causes of variance in risk

For the point of illustration, consider the classic twin model which makes the ‘equal environments assumption’ that the non-genetic effects shared by twins are the same for both MZ and DZ pairs. This assumption maximizes the proportion of familial variance attributed to genetic factors.

Suppose that the variance in risk can be decomposed into an additive genetic component (A) and a shared environment component (C) as described in Background. The risk score represents germline genetic factors for which r_rel_ can be modelled in terms of the kinship coefficients following Fisher,^1^ and the effects of non-genetic factors shared by twins can be modelled in various ways; see below.

For monozygotic (MZ) twin pairs, r_rel_ = 1. For dizygotic (DZ) twin and sibling pairs:


(9) $$r_{\text{rel}}=\left(0.5A+C\right)/\left(A+C\right).$$

This model can be extended to other relatives.^3^

The shared environmental variance component, C, can be modelled perhaps more informatively by taking into account the extent to which pairs of relatives cohabit, have cohabited or have lived apart.^6^ Non-genetic effects shared by parents and offspring,^20–24^ spouse associations^3^^,^^23^^,^^24^ and variations that take into account the birth order can be modelled.^25^^,^^26^ Despite evidence that shared environment has different roles for different types of relatives, even for those of the same degree of genetic relationship,^3^^,^^21^^,^^22^ this more nuanced modelling has not been popular among genetic researchers. Recently, we analysed epigenetic data for twins and family from across the lifespan and found evidence for non-genetic factors that would otherwise have been attributed to genes.^23^^,^^24^ Given familial aggregation is highly age-dependent, at least for breast cancer,^27^^,^^28^ it is also important to consider age and cohabitation aspects of both A and C.

### Combining risk factors

For two factors whose risk associations are virtually independent, in that their individual risk gradients Δ_i_ (i = 1,2) are essentially the same whether they are fitted alone or together, let Δ_12_ be their combined risk gradient when they are fitted together. Then:


(10) $$\Delta_{12}∼\;{\left(\Delta_{1}{}^{2}+\Delta_{2}{}^{2}\right)}^{0.5}.$$

An exact and more general formula for $\Delta_{12}$ is given in the Supplementary Material where its validity is shown for a special case.

Heuristic justification for the approximate formula comes from interpretation of Δ as the difference between cases and controls in mean risk score. If two (uncorrelated) risk scores are combined, the distance in means in two-dimensional space is the hypotenuse of a right-angled triangle whose sides are the differences in means for each of the risk scores. This argument can be extended to *n* > 2 independent risk factors in which case:


(11) $$\Delta_{1\ldots n}∼\;{\left(\Delta_{1}{}^{2}+\cdots +\Delta_{n}{}^{2}\right)}^{0.5}.$$

If the two risk scores are not acting independently (i.e. their associations are correlated) their combined associations would be attenuated, as would the third side of a less than right-angled triangle; see Supplementary Material. Therefore, the risk variance for a combination of independent risk scores, Δ_1…n_^2^, is approximately the sum of the variances of the independent components, Δ_i_^2^. This variance will be attenuated if the risk scores capture some risk factor information in common, which can also be overcome by using the OPERA concept.

### Application

As in Hopper and Carlin^11^ we study female breast cancer, but model variance in age-specific log(incidence).

### Unmeasured familial factors

First, we consider unmeasured familial factors by analysing twin associations estimated by the Nordic Twin Study,^28^ which takes into account temporal and censored aspects lacking in an earlier publication.^29^

Column two of Table 2 shows that the FRR for MZ pairs decreases from 5.91 before age 50 years to 2.50 by age 80 years. Column four shows that, given r_rel_ = 1 for MZ pairs and Equation (5), the maximum variance decreases from log(5.91) = 1.78 to log(2.50) = 0.92.

**Table 2:** Familial relative risk (FRR), twin pair covariance in log(incidence), additive genetic (A) and shared environmental (C) components of variance in log(incidence), and maximum area under the receiver operating characteristics curve from knowing all genetic causes (AUC_max_) based on data from the Nordic Twin Study of Breast Cancer^28^

Age (years)	FRR MZ	FRR DZ	Covariance MZ	Covariance DZ	A	C	AUC_max_
<50	5.91	3.51	1.78	1.26	1.04	0.74	0.83
50–59	4.93	2.77	1.60	1.02	1.15	0.44	0.81
60–69	2.98	2.24	1.09	0.81	0.57	0.52	0.77
70–79	2.5	1.8	0.92	0.59	0.66	0.26	0.75

MZ, monozygotic twin pairs; DZ, dizygotic twin pairs.

Under the classic twin model and using Equation (8), column six shows that the additive genetic variance (A) decreases with age from 1.04 to 0.66, and column seven shows that the shared environment variance (C) decreases from 0.74 to 0.26. Therefore, on average about two-thirds of the declining familial variance is attributed to genetic factors irrespective of age.

### Measured familial factors

#### Genomic risk factors

**Figure 4:**
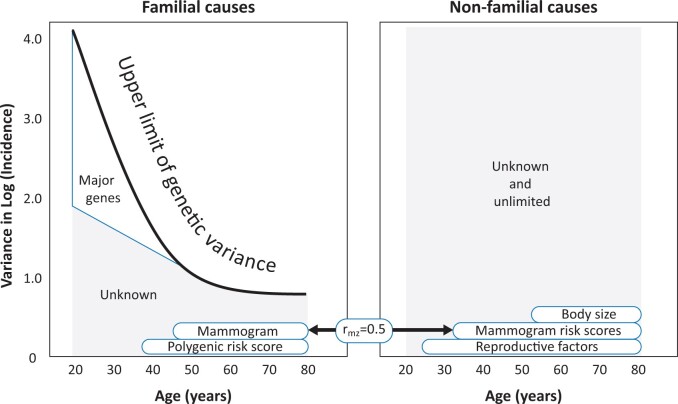
Decomposition of variance in log(incidence) of breast cancer by age according to familial effects, including rare high-risk variants in major genes such as *BRCA1* and *BRCA2*, polygenic risk scores, mammogram risk scores which have a substantial familial component and some other epidemiological risk factors that are mostly non-familial, based on literature cited in the text

Segregation analyses of multigenerational family data have also found that the total familial variance decreases with age. A substantial proportion of variance at young ages is explained by the major breast cancer susceptibility genes *BRCA1* and *BRCA2*, and a small proportion by other major genes including *ATM*, *PALB2* and *Tp53*.^30^ These major genes explain little variance for post-menopausal women; see Figure 4.

The OPERA for the current best breast cancer polygenic risk score (PRS) is log(1.65) = 0.50 so the variance explained is (0.50)^2^ = 0.25.^8^ This association is similar across all ages, although perhaps weaker before age 40 years. For women under the age of 50 years, a PRS based on 77 single nuclleotide polymorhisms (SNPS) did not explain any familial risk of breast cancer diagnosed before age 50 years.^31^ Therefore, much remains to be learned about the polygenic risk for breast cancers diagnosed at a young age; see Figure 4.

#### Non-genomic risk factors

Many non-genomic risk factors have been identified from questionnaire data. These include reproductive factors such as number and timing of live births and ages at menarche and menopause, as well as anthropometric factors height, and for post-menopausal women weight, which have historically been combined as body mass index. The risk gradients are modest, with OPERAs in the range of 1.005 to 1.2.^9^

Questionnaires attempt to reveal aetiologically relevant processes which, if measured more precisely, would have greater risk gradients. Almost all these non-genomic risk factors are correlated in relatives, usually only weakly. Therefore, they generate familial as well as mostly non-familial components of variance, and the Unifying Equation (4) describes how these are apportioned.

#### Familial aspects of non-genomic risk factors

As an example, multiple mammogram risk scores (MRSs) based on different aspects of a mammogram are being found to be associated with breast cancer risk. These include conventional mammographic density, mammographic density measured at high brightness pixel thresholds,^16^^,^^32–38^ and textural features and other agnostic measures learned by machine learning.^18^^,^^39^^,^^40^

The correlation in the MRSs based on mammographic density is about 0.6 for MZ pairs and 0.3 for DZ and sister pairs.^41^^,^^42^ The risk gradient for an MRS based on conventional mammographic density has an OPERA of about 1.5, so the variance is about 0.16, of which 0.10 would be familial and 0.06 non-familial. The risk gradient is greater for the new MRS, and when combined could be as high as 2.1,^37^ in which case the variance would be 0.55. If the MZ twin pair correlations of these new MRS are similar to those for conventional density,^42^ they could explain as much if not more familial variance than the current PRS.

#### Non-genomic non-familial risk factors

Most variation in questionnaire-based risk factors is individual-specific and makes minimal contribution to familial variance; see Figure 4. Greater specificity of exposures will increase the variance due to known non-familial factors, as is being found with the new MRS being discovered by applying artificial intelligence to digital mammography.^34^ Application to epigenetics might reveal new and mostly individual-specific risk factors.^23^^,^^24^^,^^43^

### Combinations of risk factors: independence and interactions

In general, the risk associations for known risk factors (i.e. relative risks for women of the same age) do not change greatly when fitted together; in epidemiological parlance, these associations are said to be ‘independent’ because they are additive on a particular scale. But this can be misleading. Given epidemiological analyses use the log or logit scales, a ‘lack of interaction’ on those scales means the associations of risk factors tend to multiply on one another on the absolute risk scale, on which there must be ‘interactions’ because the greater a woman is on one risk factor, the greater is her absolute risk gradient on another risk factor.^44^^,^^45^

### Combining polygenic risk scores with risk scores based on family history

Polygenic risk scores are familial, so their (relative) risk associations will not necessarily be independent of family history associations. We constructed a continuous familial risk score (FRS)^46^ from multigenerational family history data using, for example, the BOADICEA pedigree-based model.^47^ We estimated risk associations with and without fitting an established PRS and found that, for breast cancer diagnosed before age 50 years, the FRS and PRS were not correlated and their risk associations were independent. That is, the PRS discovered using mostly samples of post-menopausal women explains at most a small proportion of why breast cancer diagnosed at a young age runs in families. Figure 4 shows that the major genes and other factors dwarf the contribution of the PRS to familial risk variance in this younger age range.^30^

### Combining mammographic risk scores with polygenic risk scores

We originally predicted that ∼10% of the familial variance of breast cancer is explained by familial aspects of mammographic density (adjusted for age and body mass index).^48^ This was corroborated by estimating the change in family history associations after adjusting for this MRS.^49^ About the same proportion of SNPs associated with breast cancer have been found to be nominally associated with this MRS,^50^ but the current best PRS SNPs is at best only weakly correlated with this familial MRS.^51^^,^^52^

### Conclusion

For any risk factor, once appropriately converted into a multiplicative risk score, its ability to differentiate cases from controls is dictated by the risk gradient, log(OPERA), the square of which is the variance in risk. The familial aspects of variance can be estimated from the familial odds ratio using the Unifying Equation. The familial risk variance can be decomposed into genetic and non-genetic components by returning to Fisher’s seminal 1918 paper that converted familial correlations into variance components; see Supplementary Material or a discussion of the historical context. VALID converts familial risk ratios into variance components of risk for familial and non-familial factors, genetic and non-genetic aspects of familial risk, genomic and non-genomic aspects of genetic risk, and familial and non-familial aspects of non-genomic risk; see Figure 4.

VALID is underpinned by the OPERA concept^9^ and the key metric is Δ = log(OPERA), a natural risk gradient for a risk score. Δ can be interpreted as the difference between cases and controls in their mean risk score and is the standard deviation of log(incidence).

VALID extends the concept of ‘polygenic’ variance in risk^12^^,13^ to all other causes and can be applied to major genes by estimating the proportion of polygenic variance explained after fitting the effects of rare high-risk mutations.^30^ VALID allows the familial variance to be due to more than genetic factors alone, for example using Equation (9).

**Figure 5:**
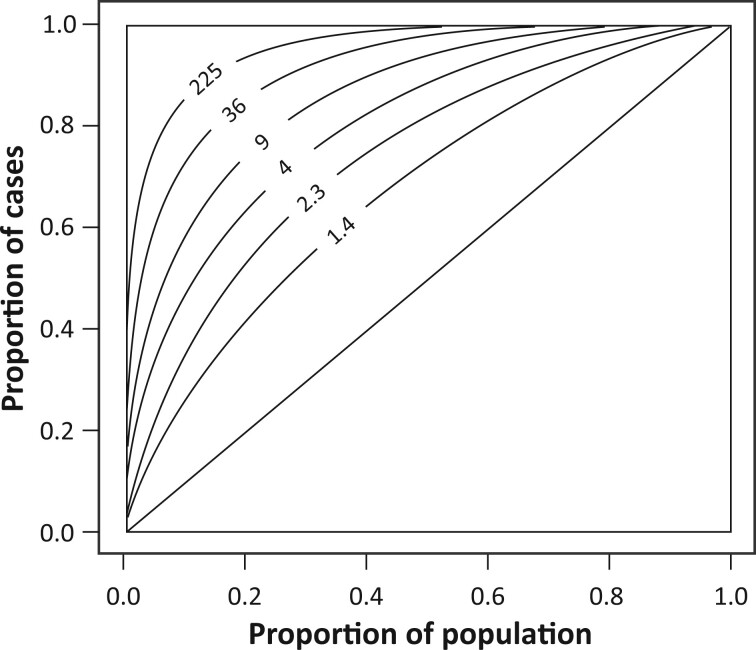
Receiver operating characteristic curves under the VALID model labelled according to the familial risk ratio (FRR), where Proportion of cases is the sensitivity and the Proportion of population is 1—specificity (following Clayton^13^), for area under the receiver operating characteristic curve (AUC) ranging 0.60, 0.73, 0.80, 0.92, 0.97 and 0.99 from lower right to upper left

Table 1 allows comparisons of the risk-discriminatory strengths of risk factors, measured and unmeasured. Note we are considering variation in risk for persons of the same age. Therefore, it is inappropriate to compare, for example, AUCs derived from cohorts estimating absolute risk for diseases whose incidence is age-dependent—particularly when this age-dependence is not necessarily universal—with AUCs derived from case-control studies. Figure 5 shows the receiver operating characteristic curve according to the FRR.

### Why log(incidence)?

Log(incidence) is a natural risk scale in epidemiology, and typically is highly dependent on age. The linearity or otherwise of its relationship to log(age) has been used to make biologically relevant inference about underlying stages in disease progression with application to common cancers^53^ and about the role of cumulative exposure to ovarian hormones in breast cancer risk.^54^

A major focus of epidemiology is on the causes of differences in log(incidence) between groups of the same age and the estimation of the risk gradients such as relative risk, odds ratio and hazard ratio by applying logistic regression to case-control studies or Cox proportional hazards regression to cohort studies. Variation in log(incidence) is the basis of complex segregation analyses of pedigree data in search of evidence for, and about, major genes.^47^ Genome-wide association studies are applying case-control analyses to create additive polygenic risk scores on this scale.^8^

### Generality

We allow the risk score to be measured or unmeasured. Whereas our multiplicative model might not accurately represent reality for every risk factor, as a model for studying combined risk factors by, for example, familial versus non-familial or genetic versus non-genetic, we illustrated how it might be a useful approximation to reality based on empirical evidence, at least for breast cancer. The model also applies to combinations of risk scores.

### Comparison with liability model and heritability

Application of the deterministic liability model to the Nordic Twin Study^28^ suggested that the influence of genetic factors on variation in risk, as measured by the tetrachoric correlation and heritability, is relatively stable with age. This is contrary to our findings from applying VALID. We think the discrepancy is explained because the AUC under the liability model is dependent on the disease prevalence as well as the tetrachoric correlation,^55^ whereas under the VALID model it depends solely on the FRR.

**Figure 6:**
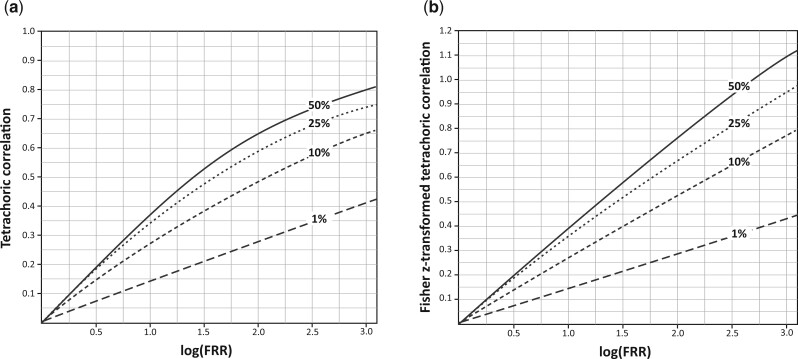
Plot of the relationship of: (a) the tetrachoric correlation calculated using the polycor package in R, and (b) the Fisher Z transform of the tetrachoric correlation, against the logarithm of the familial risk ratio (log(FRR_MZ_)) under the VALID model for disease frequencies 1%, 10%, 25% and 50%

Figure 6a shows that the relationship between the tetrachoric correlation and log(OPERA) is almost linear for log(OPERA) <1, but not thereafter, and depends highly on the disease prevalence. Figure 6b shows that on a natural scale for correlations, the Z-transformed tetrachoric correlation is almost linear with log(OPERA) across unbounded scales, though the slope still depends highly on disease prevalence.

There are two important consequences. First, for diseases with a <2-fold increased risk from having an affected first-degree relative, decomposition of familial risk will be similar whether the liability or VALID models are used. Second, the liability model predicts that the role of genes is greater for more common diseases with the same FRR, and for older persons even if the FRR is independent of age, contrary to the prediction of the VALID model and empirical evidence.

### Summary

In conclusion, we propose thinking about how risk factors explain variation in risk in terms of variance in the logarithm of age-specific incidence. Genetic and non-genetic factors combine to explain greater amounts (not proportions) of variation in risk. VALID describes the finite genetic architecture and unlimited environmental landscape of disease risk using a single metric, enabling causes of risk variation (not causes per se) to be compared and combined.

The maximum variation in risk due to genetic factors is determined by studying MZ twin pairs. Genetic factors will not be important for population risk stratification if the MZ twin pair odds ratio is weak, irrespective of disease frequency. The familial odds ratio is directly related to the absolute familial variance by the Unifying Equation. This harks back to Fisher’s 1918 paper^1^ where he showed that the major issue for evolution was the magnitude of the genetic variance, not a percentage or proportion of the total variance which he described as a ‘hotch-potch’ of a denominator.^2^ For risk, the denominator is in effect unlimited.

Our application of VALID to female breast cancer revealed that, whereas substantial components of variation in familial risk have been discovered, there remains much to be learned about the familial causes of breast cancer particularly for young women, and little is known about individual-specific variance in risk.

## Ethics approval

This study does not need ethical approval as it uses only data from published analyses.

## Supplementary Material

dyad086_Supplementary_Data

## Data Availability

Not applicable.
