# Supplementary material for: Variance of age-specific log incidence decomposition (VALID): a unifying model of measured and unmeasured genetic and non-genetic risks
Source: Int J Epidemiol. 2023 Jun 22;52(5):1557–68. doi: 10.1093/ije/dyad086 (PMC10655167; doi:10.1093/ije/dyad086)
Supplement: dyad086_Supplementary_Data [file dyad086_supplementary_data.pdf]

Supplementary Material

Historical context

Fisher's seminal 1918 paper[1] that introduced the concept of unmeasured genetic and non-genetic causes of variation in measured quantitative outcomes (traits). Fisher studied quantitative traits, measured associations between relatives using the Pearson correlation coefficient, and partitioned the variance into genetic and non-genetic components that had different and statistically independent mechanistic origins.

Briefly, Fisher assumed a trait to be the sum of independent random variables representing additive and dominance genetic components and an individual-specific (i.e. non-familial) component. He showed that, under basic assumptions, the genetic variance (not necessarily the genetic correlation) would be passed to future generations and thereby maintained in the population, a fundamental step in reconciling Mendelian inheritance for qualitative traits such as those measured by Mendel with the genetic inheritance of continuous traits such as height as measured by Galton.

In effect, Fisher converted familial correlations into variances. He pointed out that the variances of independent random variables add so that, for a set of independent causes, the absolute variance attributable to each cause will be the same whether it is considered with or without considering the other causes. He demonstrated this by decomposing familial variance into two genetic components. Others have since expanded on this approach to further decompose familial variance into genetic and non-genetic components; see below.

Fisher disliked the concept of *heritability*, the genetic variance as a proportion of total variance, and referred to it as having a "hotch-potch" of a denominator.[2] For him, the important issue was the absolute magnitude of the genetic component of variance, not its value as a

percentage. He warned that heritability can be misleading, as we found when studying large sibships born on the Greek island of Levkada, for which a substantial proportion had migrated to Australia. We found that migration was associated with an increase in non-genetic variance in blood pressure for males only.[3] Therefore, while the genetic component of variance was the same for both sexes irrespective of where they lived, the males living in Australia had a lower heritability solely because they had a larger environmental variance.

Elston and Stewart elegantly demonstrated how pedigree data on families of different size and structure could be analysed using likelihood theory.[4] Lange and colleagues expanded this to analyse quantitative data under the assumption of multivariate normality,[5] and Hopper and Mathews demonstrated the flexibility of that approach to include measured genes and take into account the role of non-genetic factors related to cohabititation.[6-7]

Combining risk scores

We prove a formula showing how the log(OPERA)s of two risk factors combine (for definition of OPERA, see Hopper[8]). We write ℓ_i instead of Δ_i for the log(OPERA) of a variable X_i in this section.

Let $X_1, \cdots, X_d$ be real-valued random variables with finite second moments and $\mathbb{E}[X_i] = 0$ for each $i = 1, \cdots, d$. Suppose that Y is a Bernoulli random variable whose conditional distribution, given $X = x$, satisfies the logistic regression equation

$$\log(odds) = \log\left(\frac{\mathbb{P}(Y = 1|X = x)}{\mathbb{P}(Y = 0|X = x)}\right) = \alpha + \beta_1 x_1 + \cdots + \beta_d x_d$$

for some real constants $\alpha, \beta_1, \cdots, \beta_d$, where $X = (X_1, \cdots, X_d)$ and $x = (x_1, \cdots, x_d)$. Note that this equation determines the conditional distribution of Y given $X = x$.

The odds ratio (OR) per standard deviation increase in X_1 is $\exp(\beta_1 SD_1)$, where SD_1 is the standard deviation of X_1 (i.e. $SD_1 = \sqrt{\mathbb{E}[X_1^2]}$, since $\mathbb{E}[X_1] = 0$). The standard deviation often gives a convenient scale for variables, so ORs are frequently reported as ORs per standard deviation. However, as noted,[1] β_1 is the log(OR) for X_1 adjusted for the other covariates, so β_1 roughly measures the effect on Y of only the part of X_1 that is not correlated with the other covariates. The standard deviation SD_1 of X_1 measures the total spread of X_1, not the spread of this relevant part of X_1. Therefore, when discussing the effect of X_1 on Y, a more natural scale for X_1 is the adjusted standard deviation ASD_1 of X_1, which we define to be the standard deviation of the part of X_1 that is uncorrelated with the other covariates $X_2, \cdots, X_d$. More precisely,

$$ASD_1 = \sqrt{\mathbb{E}[(X_1 - \pi_{2\cdots d} X_1)^2]},$$

where $\pi_{2\cdots d} X_1 = a_2 X_2 + a_3 X_3 + \cdots + a_d X_d$ and $a_2, \cdots, a_d$ are the real numbers given by

$$\begin{bmatrix} a_2 \\ \vdots \\ a_d \end{bmatrix} = \begin{bmatrix} \mathbb{E}[X_2^2] & \cdots & \mathbb{E}[X_2 X_d] \\ \vdots & \ddots & \vdots \\ \mathbb{E}[X_d X_2] & \cdots & \mathbb{E}[X_d^2] \end{bmatrix}^{-1} \begin{bmatrix} \mathbb{E}[X_1 X_2] \\ \vdots \\ \mathbb{E}[X_1 X_d] \end{bmatrix},$$

so that $\pi_{2\cdots d} X_1$ is the orthogonal projection of X_1 onto the linear span of $X_2, \cdots, X_d$ with respect to the L^2 inner product $(U, V) \longmapsto \mathbb{E}[UV]$. Then the OR per adjusted standard deviation (OPERA) for X_1 is $\exp(\beta_1 ASD_1)$, and we write ℓ_1 for the log(OPERA) of X_1, i.e. $\ell_1 = \beta_1 ASD_1$. Similarly, we can define the adjusted standard deviation ASD_2 for X_2, and we write $\ell_2 = \beta_2 ASD_2$ for the log(OPERA) of X_2.

We can also define the OPERA of the combination of X_1 and X_2 to be the OPERA of the variable $W = \beta_1 X_1 + \beta_2 X_2$, where this OPERA is defined with respect to the covariates $W, X_3, \cdots, X_d$ and the conditional distribution of Y, given $W = w, X_3 = x_3, \cdots, X_d = x_d$, that satisfies

$$\log(odds) = \alpha + w + \beta_3 x_3 + \cdots + \beta_d x_d.$$

It then follows from the above definitions that the adjusted standard deviation ASD_{12} of W is

$$ASD_{12} = \sqrt{\mathbb{E}[(W - \pi_{3\cdots d}W)^2]},$$

where $\pi_{3\cdots d}$ is the orthogonal projection onto the linear span of $X_3, \cdots, X_d$ (similar to $\pi_{2\cdots d}$, above). Then since the coefficient of w in the log-odds equation is 1, the log(OPERA) of W is ASD_{12}. Hence, by definition, the log(OPERA) ℓ_{12} of X_1 and X_2 combined is $\ell_{12} = ASD_{12}$.

We can now prove a relationship between the separate log(OPERA)s ℓ_1 and ℓ_2 of X_1 and X_2 (respectively) and the log(OPERA) ℓ_{12} of their combination. Namely, there is some $\rho \in [-1,1]$, given explicitly below, so that

$$\ell_{12} = \sqrt{\frac{\ell_1^2 + \ell_2^2 + 2\rho\epsilon\ell_1\ell_2}{1-\rho^2}}$$

whenever $\rho \neq \pm 1$, where $\epsilon = sign(\beta_1\beta_2)$ and $sign(x)$ is -1, 0 or 1 if x is strictly negative, zero or strictly positive (respectively).

Proof. By assumption, all random variables have finite second moments, so they lie in the Hilbert space L^2 whose inner product is $(U, V) \longmapsto \mathbb{E}[UV]$. The geometric notions in the following proof are all with respect to this inner product, e.g. we say two random variables U and V in L^2 are orthogonal (or perpendicular) whenever their inner product $\mathbb{E}[UV]$ is zero. Note that when U and V have zero mean, like most of the random variables below, then their inner product is equal to their covariance, so U and V are uncorrelated if and only if they are orthogonal.

Now, let $Z_1 = \beta_1(X_1 - \pi_{3\cdots d}X_1)$, $Z_2 = \beta_2(X_2 - \pi_{3\cdots d}X_2)$ and

$$Z_3 = \beta_3X_3 + \cdots + \beta_dX_d + \beta_1\pi_{3\cdots d}X_1 + \beta_2\pi_{3\cdots d}X_2,$$

so that Z_1 and Z_2 are both perpendicular to (i.e. uncorrelated with) each of $X_3, \cdots, X_d, Z_3$ and

$$Z_1 + Z_2 + Z_3 = \beta_1X_1 + \cdots + \beta_dX_d.$$

(We will later see that ρ from the statement is the correlation between Z_1 and Z_2.) We claim that, up to a sign change, ℓ_1, ℓ_2 and ℓ_{12} are equal to the log(OPERA)s of Z_1, Z_2 and their combination (respectively), where these OPERAs are with respect to the covariates Z_1, Z_2, Z_3 and the conditional distribution of Y, given $Z_1 = z_1, Z_2 = z_2, Z_3 = z_3$, that satisfies

$$\log(odds) = \alpha + z_1 + z_2 + z_3.$$

We will first prove this claim, then we will show that the log(OPERA)s of Z_1, Z_2 and their combination satisfy the desired equation. Let $\|\cdot\|$ be the norm corresponding to the inner product on L^2, so that $\|U\| = \sqrt{\mathbb{E}[U^2]}$ for any U in L^2 and so $ASD_1 = \|X_1 - \pi_{2\cdots d}X_1\|$. Also note that

$$\pi_{2\cdots d}(\pi_{3\cdots d}X_1) = \pi_{3\cdots d}X_1$$

because $\pi_{3\cdots d}X_1$ lies in the linear span of $X_3, \cdots, X_d$ and hence it lies in the linear span of $X_2, \cdots, X_d$, on which $\pi_{2\cdots d}$ is the identity. Therefore

$$\begin{aligned}\ell_1 = \beta_1 ASD_1 = \beta_1\|X_1 - \pi_{2\cdots d}X_1\| &= \beta_1\|(X_1 - \pi_{3\cdots d}X_1) - \pi_{2\cdots d}(X_1 - \pi_{3\cdots d}X_1)\| \\ &= sign(\beta_1)\ \|\beta_1(X_1 - \pi_{3\cdots d}X_1) - \pi_{2\cdots d}\beta_1(X_1 - \pi_{3\cdots d}X_1)\| \\ &= sign(\beta_1)\ \|Z_1 - \pi_{2\cdots d}Z_1\|.\end{aligned}$$

Now, the linear spans of $X_2, \cdots, X_d$ and $Z_2, X_3, \cdots, X_d$ coincide (assuming $\beta_2 \neq 0$), so $\pi_{2\cdots d}Z_1$ is the orthogonal projection of Z_1 onto the linear span of $Z_2, X_3, \cdots, X_d$. Then the formula for $a_2, \cdots, a_d$ above, but with Z_1 and Z_2 in place of X_1 and X_2 (respectively), gives $a_2 = \mathbb{E}[Z_1 Z_2]/\mathbb{E}[Z_2^2]$ and all other $a_i = 0$, since Z_1 and Z_2 are both perpendicular to $X_3, \cdots, X_d$ (directly from their definition). Therefore $\pi_{2\cdots d}Z_1 = (\mathbb{E}[Z_1 Z_2]/\mathbb{E}[Z_2^2])\ Z_2$, so

$$\ell_1 = sign(\beta_1)\left\|Z_1 - \left(\frac{\mathbb{E}[Z_1 Z_2]}{\mathbb{E}[Z_2^2]}\right) Z_2\right\|.$$

But $(\mathbb{E}[Z_1 Z_2]/\mathbb{E}[Z_2^2])\, Z_2$ is the orthogonal projection of Z_1 onto Z_2, and this is the same as the orthogonal projection of Z_1 onto the linear span of Z_2 and Z_3, since Z_1 and Z_2 are both perpendicular to Z_3. So ℓ_1 is $sign(\beta_1)$ times the log(OPERA) of Z_1, as claimed.

The proof that ℓ_2 is the log(OPERA) of Z_2 is essentially the same. The proof that ℓ_{12} is the log(OPERA) of the combination of Z_1 and Z_2 is similar but easier:

$$\ell_{12} = ASD_{12} = \|W - \pi_{3\cdots d} W\| = \|(\beta_1 X_1 + \beta_2 X_2) - \pi_{3\cdots d}(\beta_1 X_1 + \beta_2 X_2)\|$$

$$= \|\beta_1(X_1 - \pi_{3\cdots d} X_1) + \beta_2(X_2 - \pi_{3\cdots d} X_2)\| = \|Z_1 + Z_2\|,$$

which is the log(OPERA) of the combination of Z_1 and Z_2, since Z_1 and Z_2 are both perpendicular to Z_3. Therefore, the claim is proved.

Now, let ρ be the correlation between Z_1 and Z_2, i.e.

$$\rho = \frac{\mathbb{E}[Z_1 Z_2]}{\sqrt{\mathbb{E}[Z_1^2]\mathbb{E}[Z_2^2]}}.$$

Then

$$\ell_1^2 = \left\| Z_1 - \left(\frac{\mathbb{E}[Z_1 Z_2]}{\mathbb{E}[Z_2^2]}\right) Z_2 \right\|^2 = \mathbb{E}\left[\left(Z_1 - \left(\frac{\mathbb{E}[Z_1 Z_2]}{\mathbb{E}[Z_2^2]}\right) Z_2\right)^2\right]$$

$$= \mathbb{E}[Z_1^2] + \left(\frac{\mathbb{E}[Z_1 Z_2]}{\mathbb{E}[Z_2^2]}\right)^2 \mathbb{E}[Z_2^2] - 2\left(\frac{\mathbb{E}[Z_1 Z_2]}{\mathbb{E}[Z_2^2]}\right)\mathbb{E}[Z_1 Z_2] = \mathbb{E}[Z_1^2](1-\rho^2)$$

and similarly $\ell_2^2 = \mathbb{E}[Z_2^2](1-\rho^2)$. Therefore

$$\ell_{12}^2 = \|Z_1 + Z_2\|^2 = \mathbb{E}[(Z_1 + Z_2)^2] = \mathbb{E}[Z_1^2] + \mathbb{E}[Z_2^2] + 2\mathbb{E}[Z_1 Z_2]$$

$$= \mathbb{E}[Z_1^2] + \mathbb{E}[Z_2^2] + 2\rho\sqrt{\mathbb{E}[Z_1^2]\mathbb{E}[Z_2^2]} = \frac{\ell_1^2 + \ell_2^2 + 2\rho\sqrt{\ell_1^2 \ell_2^2}}{1-\rho^2}$$

$$= \frac{\ell_1^2 + \ell_2^2 + 2\rho\ell_1\ell_2\, sign(\ell_1\ell_2)}{1-\rho^2} = \frac{\ell_1^2 + \ell_2^2 + 2\rho\ell_1\ell_2\, sign(\beta_1\beta_2)}{1-\rho^2}$$

which completes the proof, since $\ell_{12} \geq 0$. ■

We finish by calculating ρ in terms of the covariances $c_{ij} = \mathbb{E}[X_i X_j]$. Note that the correlation $Corr(U,V)$ between random variables U and V in L^2 is invariant under a positive rescaling of the variables, so

$$\rho = Corr(Z_1, Z_2) = Corr\big(\beta_1(X_1 - \pi_{3\cdots d}X_1), \beta_2(X_2 - \pi_{3\cdots d}X_2)\big)$$

$$= sign(\beta_1\beta_2)\, Corr\big((X_1 - \pi_{3\cdots d}X_1), (X_2 - \pi_{3\cdots d}X_2)\big)$$

$$= \frac{\epsilon\, \mathbb{E}[(X_1 - \pi_{3\cdots d}X_1)(X_2 - \pi_{3\cdots d}X_2)]}{\sqrt{\mathbb{E}[(X_1 - \pi_{3\cdots d}X_1)^2]\mathbb{E}[(X_2 - \pi_{3\cdots d}X_2)^2]}},$$

where $\epsilon = sign(\beta_1\beta_2)$, as above. We will now calculate the expectations in the right-hand side of this equation. First note that

$$\pi_{3\cdots d}X_1 = \sum_{i=3}^{d} p_i X_i \quad \text{and} \quad \pi_{3\cdots d}X_2 = \sum_{j=3}^{d} q_j X_j$$

where $p_3, \cdots, p_d$ and $q_3, \cdots, q_d$ are real numbers which can be given in terms of the covariances $c_{ij} = \mathbb{E}[X_i X_j]$ by expressions similar to the one for $a_2, \cdots, a_d$, above. Explicitly,

$$\begin{bmatrix} p_3 \\ \vdots \\ p_d \end{bmatrix} = \begin{bmatrix} c_{33} & \cdots & c_{3d} \\ \vdots & \ddots & \vdots \\ c_{d3} & \cdots & c_{dd} \end{bmatrix}^{-1} \begin{bmatrix} c_{13} \\ \vdots \\ c_{1d} \end{bmatrix} \quad \text{and} \quad \begin{bmatrix} q_3 \\ \vdots \\ q_d \end{bmatrix} = \begin{bmatrix} c_{33} & \cdots & c_{3d} \\ \vdots & \ddots & \vdots \\ c_{d3} & \cdots & c_{dd} \end{bmatrix}^{-1} \begin{bmatrix} c_{23} \\ \vdots \\ c_{2d} \end{bmatrix}.$$

For convenience, we define $p_1 = -1, p_2 = 0$ and $q_1 = 0, q_2 = -1$. Then

$$\mathbb{E}[(X_1 - \pi_{3\cdots d}X_1)(X_2 - \pi_{3\cdots d}X_2)] = \mathbb{E}\left[\left(X_1 - \sum_{i=3}^{d} p_i X_i\right)\left(X_2 - \sum_{j=3}^{d} q_j X_j\right)\right]$$

$$= \mathbb{E}\left[\left(\sum_{i=1}^{d} p_i X_i\right)\left(\sum_{j=1}^{d} q_j X_j\right)\right] = \sum_{i,j=1}^{d} p_i q_j \mathbb{E}[X_i X_j] = \sum_{i,j=1}^{d} p_i q_j c_{ij} = p^T C q,$$

where $p = (p_1, \cdots, p_d)$, $q = (q_1, \cdots, q_d)$ and C is the matrix whose $(i,j)^{th}$ element is c_{ij}. The other two expectations can be calculated similarly, so we obtain

$$\rho = \frac{\epsilon(p^T C q)}{\sqrt{(p^T C p)(q^T C q)}}.$$

Example. Suppose that $d = 3$ (so there is only one adjustment variable X_3) and that X_1, X_2 and X_3 are all standardised (to have mean 0 and marginal standard deviation 1). Then the above formula for ρ becomes

$$\rho = \frac{\rho_{12} - \rho_{13}\rho_{23}}{\sqrt{(1-\rho_{13}^2)(1-\rho_{23}^2)}}$$

where ρ_{ij} is the correlation between X_i and X_j. If X_1 and X_2 are uncorrelated ($\rho_{12} = 0$) then we need $\rho_{13}^2 + \rho_{23}^2 < 1$ so that the variance-covariance matrix of (X_1, X_2, X_3) is positive definite. The exact formula for ℓ_{12} above shows that

$$\ell_{12} \approx \sqrt{\ell_1^2 + \ell_2^2}$$

if $\rho \approx 0$. Numerical calculations show that exact formula for ℓ_{12} is between 0% and 11% higher than this approximate formula, whenever the correlations ρ_{13} and ρ_{23} are both between -0.4 and 0.4. Therefore, the approximate formula for ℓ_{12} is a good approximation for uncorrelated risk factors X_1 and X_2 that are not strongly correlated with the adjustment variable X_3.

Relationship between AUC and Δ = log(OPERA)

For a continuous risk factor X and a binary outcome Y, under a model where X is normally distributed in both cases and controls, we prove that:

$$AUC = \Phi(\Delta/\sqrt{2})$$

where

$$OPERA = \exp(\Delta),$$

Φ is the cumulative distribution function of the standard normal distribution,

Let X and Y be random variables representing a continuous risk factor and the affected status of a person randomly selected from a given population, respectively. We suppose that $Y = 1$ corresponds to a case and $Y = 0$ corresponds to a control, and we let $q_1 = P(Y = 1)$ be the prevalence of disease, so that $q_0 + q_1 = 1$ where $q_0 = P(Y = 0)$. We assume the distribution of the risk factor X for cases is $N(\Delta, 1)$ (i.e., the conditional distribution of X given $Y = 1$ is the univariate normal distribution with mean Δ and variance 1) and the distribution of X for controls is $N(0,1)$; see Wentzensen and Wacholder.[9]

The area under the (receiver operating characteristic) curve, AUC, is the probability that a randomly selected case will have a higher value of the risk factor than a randomly selected control. Suppose we randomly (and uniformly) select a control and a case from the population, and let X_0 and X_1 be their risk factors, respectively. Then X_0 and X_1 are independent random variables, and under the above model they have marginal distributions $X_0 \sim N(0,1)$ and $X_1 \sim N(\Delta, 1)$.

Therefore $X_1 - X_0 \sim N(\Delta, 2)$, so $Z = (X_1 - X_0 - \Delta)/\sqrt{2}$ is standard normal, hence

$$AUC = P(X_1 > X_0) = \mathrm{P}\left(\frac{X_1 - X_0 - \Delta}{\sqrt{2}} > -\frac{\Delta}{\sqrt{2}}\right) = \mathrm{P}\left(\mathrm{Z} > -\frac{\Delta}{\sqrt{2}}\right) = \mathrm{P}\left(\mathrm{Z} < \frac{\Delta}{\sqrt{2}}\right) = \Phi\left(\frac{\Delta}{\sqrt{2}}\right),$$

where Φ is the cumulative distribution function of the standard normal distribution.

Relationship between OPERA and Δ

We define

$$\phi_1(x) = P(X = x \mid Y = 1) = \frac{1}{\sqrt{2\pi}} \exp\left(-\frac{(x - \Delta)^2}{2}\right)$$

and similarly

$$\phi_0(x) = P(X = x \mid Y = 0) = \frac{1}{\sqrt{2\pi}} \exp\left(-\frac{x^2}{2}\right).$$

If $p_1(x)$ is the probability that a person with biomarker $X = x$ is a case, then

$$p_1(x) = P(Y = 1 \mid X = x) = \frac{P(X = x \mid Y = 1)P(Y = 1)}{P(X = x)} = \frac{q_1\phi_1(x)}{q_0\phi_0(x) + q_1\phi_1(x)},$$

since

$$P(X = x) = \sum_{y=0}^{1} P(X = x, Y = y) = \sum_{y=0}^{1} (X = x \mid Y = y)\, P(Y = y) = q_0\phi_0(x) + q_1\phi_1(x).$$

Then the probability $p_0(x)$ that a person with risk factor $X = x$ is a control is $1 - p_1(x)$, hence

$$p_0(x) = \frac{q_0\phi_0(x)}{q_0\phi_0(x) + q_1\phi_1(x)}.$$

Therefore the odds, $odds(x)$, that a person with biomarker $X = x$ is a case is

$$odds(x) = \frac{p_1(x)}{p_0(x)} = \frac{q_1\phi_1(x)}{q_0\phi_0(x)}.$$

The odds ratio $OR(x)$ for a person with risk factor $X = x + 1$ compared to a person with risk factor $X = x$ is

$$OR(x) = \frac{odds(x + 1)}{odds(x)} = \frac{\dfrac{q_1\phi_1(x + 1)}{q_0\phi_0(x + 1)}}{\dfrac{q_1\phi_1(x)}{q_0\phi_0(x)}} = \frac{\dfrac{\phi_1(x + 1)}{\phi_1(x)}}{\dfrac{\phi_0(x + 1)}{\phi_0(x)}}.$$

Using the formula for $\phi_1(x)$ above,

$$\frac{\phi_1(x + 1)}{\phi_1(x)} = \frac{\exp(-(x - \Delta + 1)^2/2)}{\exp(-(x - \Delta)^2/2)} = \exp\left(\Delta - x - \frac{1}{2}\right)$$

and similarly

$$\frac{\phi_0(x + 1)}{\phi_0(x)} = \exp\left(-x - \frac{1}{2}\right).$$

Substituting these equations into the expression for $OR(x)$ above gives

$$OR(x) = \frac{\exp\left(\Delta - \mathrm{x} - \frac{1}{2}\right)}{\exp\left(-\mathrm{x} - \frac{1}{2}\right)} = \exp(\Delta).$$

That is, the *OR* is independent of X and is equivalent to the odds ratio per standard deviation or OPERA and therefore log(OPERA) = Δ.

Relationship between IQRR and Δ

By definition, the inter-quartile risk ratio (IQQR) is

$$IQRR = \frac{P(Y = 1|X > b)}{P(Y = 1|X < a)}$$

where a and b are the 25th and 75th percentiles of X (respectively), so that they define the boundaries of the lower and upper quartiles. But we have

$$P(Y = 1|X < a) = \frac{P(X < a|Y = 1)P(Y = 1)}{P(X < a)} = 4q_1\Phi(\mathrm{a} - \Delta),$$

since: $P(Y = 1) = q_1$ by definition; $P(X < a) = 1/4$ because a is the 25th percentile of X; and the distribution of X for cases is $N(\Delta, 1)$. Similarly,

$$P(Y = 1|X > b) = 4q_1\big(1 - \Phi(\mathrm{b} - \Delta)\big) = 4q_1\Phi(\Delta - \mathrm{b}),$$

where the last step uses the fact that the standard normal distribution is symmetric. Taking the ratio of these two expressions gives

$$IQRR = \frac{\Phi(\Delta - \mathrm{b})}{\Phi(\mathrm{a} - \Delta)}.$$

To determine a and b, by the definition of a we have

$$\frac{1}{4} = P(X < a) = \sum_{i=0}^{1} P(X < a|Y = i)P(Y = i)$$

$$= q_0P(X < a|Y = 0) + q_1P(X - \Delta < a - \Delta|Y = 1) = q_0\Phi(\mathrm{a}) + q_1\Phi(\mathrm{a} - \Delta).$$

Similarly, we have

$$\frac{3}{4} = P(X < b) = q_0\Phi(\mathrm{b}) + q_1\Phi(\mathrm{b} - \Delta).$$

Given the disease prevalence q_1, these equations can be solved numerically for a and b (for any given Δ) and these can be substituted into the equation above to give the $IQRR$ as a function of Δ. If the distribution of X is approximately the distribution of the risk factor in the controls and is standard normal, a $\approx \Phi^{-1}(0.25) = -0.6745$ and b $\approx \Phi^{-1}(0.75) = 0.6745$.

References to Supplementary Material

1. Fisher RA. The correlation between relatives on the supposition of Mendelian inheritance. *Trans Roy Soc Edinburgh* 1918;**52**:399– 433.
2. Fisher RA. Limits to intensive production in animals. *Br Agric Bull* 1951;**4**:217–18.
3. Harrap SB, Stebbing M, Hopper JL et al. Familial patterns of covariation for cardiovascular risk factors in adults: The Victorian Family Heart Study. *Am J Epidemiol* 2000;**152**:704–15.
4. Elston RC, Stewart J. A general model for the genetic analysis of pedigree data. *Hum Hered* 1971;21:523-42.
5. Lange K, Westlake J, Spence MA. Extensions to pedigree analysis. Extensions to pedigree analysis. III. Variance components by the scoring method. *Ann Hum Genet* 1976;39:485-91.
6. Hopper JL, Mathews JD. Extensions to multivariate normal models for pedigree analysis. *Ann Hum Genet* 1982;**46**:373-83.
7. Hopper JL, Tait BD, Propert DN, Mathews JD. Genetic analysis of systolic blood pressure in Melbourne families. *Clin Exp Pharmacol Physiol* 1982;**9**:247-52.

8. Hopper JL. Odds per adjusted standard deviation: comparing strengths of associations for risk factors measured on different scales and across diseases and populations. Am J Epidemiol 2015;182:863-7.

9. Wentzensen N, Wacholder S. From differences in means between cases and controls to risk stratification: a business plan for biomarker development. *Cancer Discov* 2013;**3**,148–157.
